# The Microscopic Appearances of Cancer Cells

**Published:** 1871-07

**Authors:** I. N. Danforth

**Affiliations:** Chicago


ARTICLE II.—*The Microscopic Appearances of Cancer Cells.* By I. N. DANFORTH, M.D., Chicago.

Twenty years ago, the opinion was pretty generally entertained in the medical world, that the microscope, if used by skillful hands, was entirely competent to decide as to the malignancy or benignancy of any and all morbid growths. In the twenty-fifth volume of the "American Journal of the Medical Sciences," may be found a lengthy and elaborate article, evidently the product of much and careful study, in which the author, Dr. F. Donaldson, of Baltimore, sets forth the distinctive characteristics of cancer cells, and adds many figures intended to illustrate these peculiarities. Dr. Donaldson's attempts at classifying cancer cells should have convinced him, it would seem, that these cells at least, construe very liberally their obligations to conform to the requirements of a uniform law of type. He makes six varieties:

"1. The polygonal, or more or less spherical and ovoid cell.

"2. The caudated cell.

"3. The fusiform cell.

"4. The concentric cell.

"5. The compound, or mother cell.

"6. Agglomerated nuclei, connected by granular homogenous substance."

This classification must certainly have been conceived in a truly liberal spirit. It is broad-gauged enough to cover every cell in every part and organ of the body, whether in health or in disease; and not only that, but also to include every cell in every plant and animal that flourishes upon or walks over the earth, and of every inhabitant of the boundless ocean. Let any one set himself squarely down to the work of imagining what the microscopic appearance of "the polygonal, or more or less spherical and ovoid cell" must be, and he will find himself engaged in a task which is limited only by his powers of imagination. Indeed I can scarcely conceive of an exercise better calculated to develop that faculty of the mind. The fact is, Dr. Donaldson's observations, instead of proving that anything like uniformity exists in the structure and appearance of cancer cells, very clearly proved that the distinguishing characteristic of these bodies is a most perverse and constant want of uniformity, and in doing this, he made a very important and welcome addition to our knowledge of the minute structure of cancerous growths. The very active researches which nave been going on in every department of pathological histology during the last few years, have but served to buttress and substantiate the statement that *there is no typical cancer cell*, as there is a typical pus cell and a typical epithelial cell. The very nature of cancer; its comparatively rapid growth; its lawless invasion and destruction of all adjacent tissues; its short life and imperfect organization, and, moreover, its rapid decay, are, each and all, sufficient reasons for expecting many and varying types of cell growth. These several peculiarities are worth glancing at a little more closely.

*First:* Cancer is a structure of essentially feeble organizing power; in other words, it is endowed with a low grade of vital power. In its mode of growth its behavior is strikingly like the so-called "fungus" or "proud flesh," which is so apt to sprout up in wounds which do not heal kindly. Indeed, it is not perhaps far out of the way to describe it as a sort of fungus growth implanted within or engrafted upon normal tissue. Hence, like all productions of like nature, its growth is rapid as compared with healthy structures; and this very rapidity necessitates incompleteness, or a faulty and unfinished growth. Hence, also, we should expect to find cells in all stages of growth, as well as a multiplicity of cell forms. We find caudate cells, pushing out their processes where they meet with the least resistance—in other words, growing wherever they find room to grow, without, in their haste, stopping to consider the results; we find half-grown cells, looking like overgrown nuclei; large, awkward, angular cells, frequently with several nuclei, (the so-called "mother" or "brood" cells) which seem only intent upon leaving a numerous progeny behind them; cells with one or more "buds" or processes protruding from their walls, which is also a form of cell multiplication, and, finally, cells which combine two or more of these various phases of growth. Therefore, one peculiarity of cancer cells depends upon its rapidity of, as well as faulty growth.

*Secondly:* Cancer is notoriously lawless and unsparing in its progress. Unlike other morbid growths, it invades all structures which occupy its pathway, infiltrating glands, insinuating its growing germinal matter between the fibres of muscle, or actually trespassing upon the substance of the individual fibre itself; plowing its way into and through osseous tissue; perforating membranes; tapping blood-vessels; surrounding and compressing nerves; and, ultimately, destroying and disintegrating whatever it touches. But in accomplishing this work of destruction, cancer liberates very many formed and forming cells which do not properly belong to it. That is, the *normal* cells of *healthy* structure, whether it be glandular or otherwise, become admixed with the *abnormal* cells of diseased (cancerous) structure, just so fast as the latter invades and disintegrates the former. A specimen of epithelial cancer of the lip before me, shows a multitude of epithelial cells which cannot be distinguished from those of the healthy structure, and these are most abundant at or near the junction of the healthy with the diseased tissue. A specimen of encephaloid of the liver contains very many cells precisely like, and undoubtedly identical with, the secreting cells of this organ, and in both instances, the cells of health and disease lie in the most intimate relations. Here, then, we have another reason for the great variety in regard of form and size, which we so constantly meet with in the study of cancer cells.

*Thirdly:* The term of life of any given cancer cell is necessarily a brief one; its organization necessarily imperfect, or of low type, and its decay and disorganization rapid. Hence, in almost every specimen of cancer cells, we may expect to find, and, indeed *do* find, many cells in different stages of decay, and, consequently, undergoing various morphological changes. That part of the cell which is first formed is likewise first to die, and the external part, or periphery, (the so-called "formed material") is now almost universally regarded as the oldest portion of the cell; hence we find cells with angular or roughened boundaries, because the external portion is slowly or rapidly disintegrating: but sometimes large masses of the "formed material" will disappear at once, and this gives to many cells their awkward and ill-shaped appearance: others seem to wither and become shriveled, or, possibly, dessicated; hence, we find cells presenting a pinched, dried, starved appearance: others still maintain their integrity in regard of form and size, but become filled with granular matter, which is probably the product of fatty degeneration; hence we find cells which are dark or granular, or cloudy or nebulous in appearance. Again, all cancers speedily undergo—or are constantly undergoing—fatty degeneration both within and between their component cells; or perhaps it is more correct to say that this change always commences *in* the cells, but that the fat drops are shortly liberated as the result of cell-decay. Here then we find still another element of complexity—namely, the presence of many fat globules, both floating free between the proper cells, and likewise enclosed within, or forming a part of, these cells.

It need not, therefore, be a matter of surprise that cancer cells should bid defiance to any and all rules of classification. It is, indeed, rather a matter of surprise that any attempt at such a classification should ever have been made, seeing that, for all diagnostic purposes, we are far better off as we are; and, for all pathological purposes, we should not be a hairs-breadth nearer the truth regarding the real *nature* of cancer *with* a formal classification than we are *without* one.

A very important practical question, however, still presents itself, namely, Can cancer be diagnosticated with any degree of certainty, by its microscopic appearances? It is easy enough to answer this question to my own satisfaction; it is possibly less easy to make the response intelligible to those unaccustomed to the study of cell forms. I believe that the practiced microscopist can have little difficulty in satisfying himself as to the character of a morbid growth, provided always he receives the specimen in a a state fit for examination. I have myself received many specimens for examination which consisted of little bits, snipped from the surface of a morbid growth, and then rolled in a dry paper or rag, and carried in the pocket for hours afterward, until they became perfectly hardened and dry. The idea of forming a conclusion from such specimens as these is simply absurd and ridiculous. The constituent cells of such a specimen must of very necessity undergo changes of form and consistency which cannot be obviated by any artificial means; hence, any conclusions drawn from them must be, to say the least, very unreliable. A specimen intended for microscopic examination should be taken from beneath the surface of the tumor, and as near its centre as possible; it should be examined at once whenever this is practicable, and in any event, it should be kept moist until it passes to the hands of the microscopist. If these conditions be complied with, I believe that a correct conclusion can be arrived at in so large a proportion of cases, that the exceptions are not worth considering, if the examination be made by one accustomed to the study of morbid growths. Moreover, I believe that any one who possesses a microscope of fair quality, and who can use it with a reasonable amount of common sense, can satisfy himself as to whether a morbid growth is or is not cancerous in its nature. Possibly a description of cancer cells will still be expected. They are at once very easy and very difficult to describe. If the illustrations and descriptions of these cells given by Glüge, Paget, Henry H. Smith, Beale, Gross, Moore, Bennett and others, be consulted, they will be found to be all very *like* and all very *unlike*, except when the same engravings have been used by two or more different authors; and this is simply because no two illustrations representing *actual specimens* can be alike. The distinguishing characteristic of cancer cells is a *want of uniformity*, or an absolute non-conformity to any law of type—and this for reasons which I have already given. It is, indeed, true that a cancerous growth, especially in its early stages, attempts to produce cells which are more or less like those of the tissue out of which, or within which, or near which, it is differentiated, as we so often see in the case of epithelial cancer; but this attempt is never successful, even at the very beginning, and it is shortly abandoned in so far as an allegiance to typical forms is concerned. Hence this very want of uniformity in regard of cell forms, constitutes the pivot upon which the diagnosis turns when the microscope is appealed to. It is not because we *can* classify cancer cells that we *call* them such; but rather because we *cannot* classify them. It is not because they are old microscopic acquaintances that we call them malignant; but rather because they are always strangers. In fact it is just precisely this diversity of appearance that proves their malignancy; for it indicates their rapid and lawless growth, their disrespect for and destruction of adjacent tissues, and their rapid death, and these, taken together, constitute the very essence of malignancy. If fifty specimens of keloid be examined, the ultimate elements of each one will be found to be, not precisely alike—for cells are no more precisely alike than leaves or apples or men are precisely alike—but formed on the same general model as regards shape and size, and, therefore, conforming to a law of type. If fifty specimens of ordinary fatty tumor be examined, the same law will be found to hold good. Moreover, in both these cases, the morbid growth will show some respect for the rights of neighboring tissues. If, indeed, the latter are crowded out of their rightful position, they will not therefore be denied the privilege of existing somewhere else. But an examination of twice this number of cancer specimens will only the more absolutely demonstrate the fact that in place of law of type we have lawlessness of type, and that this very lawlessness is the microscopic peculiarity of cancer. Hence, in the so-called "innocent" or "benignant" growths, we are to look for a kind of multiform unity, and in malignant growths, we may, with equal confidence, expect a uniform multiformity, or an absolute non-conformity to any ideal cell type.

One other, and scarcely less important point should be noticed. Errors of *location* are generally coincident with diversity of form in cancerous growths, and this should be taken into account in establishing the diagnosis. The very fact that a mass of cells have infiltrated or invaded a tissue or organ, and that they persistently and unaccountably vary in size, form and behavior from the normal cells of such tissue or organ, is all but, and perhaps quite, sufficient to stamp them with the seal of malignancy. They are at once recognized as marauders and pirates, intent only upon the destruction of everything within their reach. To attempt to describe a specimen of cancer cells, is to huddle together as many epithets as we can lay our hands on, to pester and perplex the dictionary makers by bringing forth a new progeny of words, and appropriating them to the same use, and, after all, to fail of giving a reliable description of bodies which obstinately refuse to look twice alike. It seems to me that we best describe cancer cells when we simply say that we *cannot* describe them; when we say that they present an endless diversity of forms, and that, *therefore*, they are cancer cells.

In conclusion, I would lay down the following simple rules for drawing the distinction between innocent and morbid growths; whenever a description of *one* of the cells of a microscopic specimen is a description of *all* of its cells, the chances are as ten to one that it is *not* cancer;—whenever, on the other hand, the cells of such a specimen are so varied in form and size that philology and ingenuity and imagination, and the most unflinching resolution combined, utterly fail to accomplish the task of describing them, the chances are as ten to one that the specimen is from a malignant growth, whatever may be its name or location.

---

PILL-AGE.—Dr. Brandreth, out of his pills, has scraped together almost $2,000,000, and has life insurance policies amounting to $140,000 besides. It is thought he does not take his own pills.

*Doctor's Motto*—Patients and long suffering.

